# Microscopic Photosensitization: A New Tool to Investigate the Role of Mitochondria in Cell Death

**DOI:** 10.1100/tsw.2002.227

**Published:** 2002-05-03

**Authors:** May-Ghee Lum, Tetsuhiro Minamikawa, Phillip Nagley

**Affiliations:** Department of Biochemistry and Molecular Biology, PO Box 13D, Monash University, Victoria 3800, Australia; Olympus Australia Pty. Ltd., 1/104 Ferntree Gully Rd., Oakleigh VIC 3166, Australia

**Keywords:** apoptosis, mitochondria, photosensitization, confocal microscopy, Chloromethyl-X-Rosamine, MitoTracker Red

## Abstract

Active involvement of mitochondria in cell death has been well-documented, but local apoptotic signaling between subsets of mitochondria has been poorly explored to date. Using mitochondrially localized CMXRos as a photosensitizer coupled to laser irradiation by confocal laser scanning microscopy, we demonstrate that partial irradiation of about half the mitochondria in human 143B TK^–^ cells induces rapid loss of mitochondrial membrane potential (ΔΨm) in nonirradiated mitochondria. Cells so partially irradiated show apoptotic indications, including mobilization of cytochrome c and binding of annexin V within 2 h following irradiation. The loss of ΔΨm in nonirradiated mitochondria did not occur in cells photoirradiated in the absence of CMXRos. Increasing the proportion of irradiated mitochondria in each cell (up to about 50%) generated a correspondingly greater percentage of cells in which nonirradiated mitochondria lost ΔΨm and which also showed apoptotic indications. Only at the highest level of irradiation (global for all mitochondria in one cell) were signs of necrosis evident (judged by uptake of propidium iodide). Because laser irradiation is specific to the subpopulation of mitochondria targeted, the data imply that a signal emanating from irradiated mitochondria is processed by their nonirradiated counterparts. We conclude that intermitochondrial signaling occurs in the subcellular response to induction of apoptosis.

## INTRODUCTION

The importance of mitochondria in cell biology has been increasingly well-recognized in recent years. Mitochondria not only are powerhouses in cells but also are dynamic participants in cellular regulation, and they execute decisions critical to cell death. A key feature of the mitochondrially mediated cell death is the release of proapoptotic signaling molecules such as cytochrome *c* (cyt c) from the mitochondria into the cytosol. Cytosolic cyt c participates in a well-defined caspase-activating pathway via the apoptosome[1] to immediately activate caspase-9 and consequently turn on caspase-3, committing the cell to die. Another important feature related to cellular debilitation is a sudden induction of mitochondrial dysfunction, manifested by collapse of electrical membrane potential across the mitochondrial inner membrane ($\Delta\Psi_m$). This is due to the opening of a specific pore in that membrane[2], activating the mitochondrial permeability transition (MPT)[3].

While the basic mechanisms by which mitochondria participate in the execution of apoptosis have been defined on a cell population level[4], much remains to be investigated regarding local apoptotic signaling between subsets of mitochondria. The possibility that intermitochondrial signaling occurs during an apoptotic response arises from several observations. In a study on HeLa cells, Goldstein et al.[5] found that all mitochondria in any given cell release cyt c despite intermitochondrial heterogeneity in the timing for release of cyt c. This suggests that activation of a critical number of mitochondria by upstream death signals may be sufficient to induce cyt c release in the remainder of the mitochondria. In another study, $Ca^{2+}$-induced MPT events in one part of a myotube generated depolarization waves that propagated along myotubes and initiated caspase-dependent apoptosis[6], explicitly demonstrating lateral signaling between mitochondria.

We have previously shown that Chloromethyl-X-rosamine (CMXRos; MitoTracker Red) possesses a strong photosensitizing action and induces apoptosis in intact human cells[7]. Here we have applied microscopic photosensitization, a novel technique utilizing CMXRos as a mitochondria-selective photosensitizer and the specificity of the confocal laser-scanning beam to photoirradiate a subpopulation of mitochondria in a given cell. The aim of this study was to determine whether death signals can be transmitted from irradiated mitochondria and received by the nonirradiated mitochondria during apoptotic induction. The data revealed that microscopic photosensitization with CMXRos on a subset of mitochondria (up to about 50%) induced global $\Delta\Psi_m$ collapse in all mitochondria and cellular apoptosis associated with early cyt c release. This finding indicates that intermitochondrial signaling occurs during apoptosis, having been initiated by severe phototoxic effects in the irradiated set of mitochondria.

## MATERIALS AND METHODS

### Materials and Cell Lines

Fluorescent dyes including rhodamine 123 (Rh123), CMXRos, Annexin V conjugated to Alexa Fluor 488, and goat antimouse IgG conjugated to Alexa Fluor 488 were purchased from Molecular Probes. Propidium iodide (PI) was acquired from Sigma. Monoclonal mouse anti-cyt c IgG (clone 6H2.B4) was acquired from Pharmingen. Human osteosarcoma 143B $TK^-$ cells were cultured as previously described[8].

### Microscopic Photosensitization by Partial Irradiation

Cells were seeded the day before irradiation into 35-mm dishes at a density of $6 \times 10^4$ cells per dish, the bottom of which was fenestrated and sealed with a round-grid coverslip (CELLocate,

Eppendorf, grid size 175 μm). For photosensitization, cells were loaded with CMXRos (200 nM, 15 min) at 37°C, then washed and resuspended with fresh phenol-red-free RPMI 1640 medium. To detect $\Delta\Psi_m$ response, cells were coloaded with Rh123 (10 μM, 15 min) at 37°C. Cells were maintained at 37°C in a temperature-controlled chamber (Life Science Resources Ltd, Cambridge, U.K.) on the stage of an inverted Leica DMIRB confocal microscope (TCS-NT system). Single cells targeted for photoirradiation were initially observed under dim transmission illumination from a tungsten lamp, and the cellular position on the grid coverslip was noted. The target cell was then positioned at the center of the visual field, and an image of the whole cell was obtained at low laser intensity (zoom 1, 2-4 scans) to minimize phototoxicity. A portion of the cell (typically, half of the cell unless otherwise stated) was photoirradiated at high laser intensity to induce damage on a subpopulation of mitochondria in a given cell. Typically, partial irradiation was conducted by continuous xy scanning using the 488- and 568-nm excitation from Ar/Kr laser at 128 scans (1 s/scan), zoom 8, and imaged with a Leica PL APO 63X/NA 1.2 water immersion objective. Immediately after irradiation, the whole cell was imaged at low laser intensity (zoom 1, 2 scans), and the same cell was monitored at appropriate time points following subsequent staining with other fluorescent indicators for cell death events. Unstained cells that were partially irradiated under these conditions were able to undergo cell division more than 8 h after irradiation.

## Imaging

Fluorescent images were obtained under confocal microscopy conditions as above. Excitation wavelength/detection filter settings for each of the fluorescent indicators were as follows: CMXRos and PI, 568/665-nm longpass; Alexa Fluor 488, 488/530-nm bandpass filter. For simultaneous imaging of Rh123/CMXRos, Alexa Fluor 488/CMXRos, laser excitation used was 488 and 568 nm. Image analysis was performed with ImageTool version 2.00 (The University of Texas Health Science Center in San Antonio).

## Assessment of Apoptosis

Release of cyt c into the cytosol was assessed by immunocytochemical staining of cyt c. Cells in 35-mm dishes were fixed with 3.5% paraformaldehyde for 10 min, rinsed with PBS three times, and permeabilized with 0.1% Triton-X-100 for 10 min. This was followed by incubation with anti-cyt c monoclonal antibody (diluted 1/100) for 2 h and with secondary goat antimouse antibody (diluted 1/200) conjugated to Alexa Fluor 488 for 1 h. After each stage of incubation, cells were rinsed with PBS three times before mounting in PBS and imaged within 24 h by confocal microscopy. For tests of annexin V binding, the medium was gently removed following partial irradiation, and cold annexin-binding buffer containing annexin V-Alexa Fluor 488 conjugates (5%) was added. Cells were incubated at room temperature for 15 min followed by addition of cold annexin-binding buffer containing PI (5 μg/ml) to distinguish early apoptotic from late apoptotic or necrotic cells. Cells were then imaged by confocal microscopy.

## Quantification of Mitochondria Subjected to Irradiation

For partial irradiation, a zone of the cell corresponding to a particular proportion of mitochondria was estimated by eye and intensively scanned with the laser. The precise percentage of mitochondria irradiated was determined postacquisition of images. For this purpose, CMXRos images were obtained (a) before irradiation that depicted all mitochondria in a given cell and (b) during irradiation that shows the zone where that subset of mitochondria had been irradiated. The

CMXRos intensity of the images were adjusted manually to a threshold level (using ImageTool software) to highlight the mitochondria that appeared as black pixels against a white background. The number of black pixels was quantified separately for the respective images showing either (a) total mitochondria or (b) irradiated mitochondria. The proportion of mitochondria irradiated was thus expressed as a percentage of the total mitochondria in a given cell.

## RESULTS AND DISCUSSION

### Rapid $\Delta\Psi_m$ Loss Induced in Nonirradiated Mitochondria Following Partial Irradiation

Our previous study showed that global irradiation on all mitochondria within a given cell rapidly induces mitochondrial depolarization[7]. Here we partially irradiated a subset of mitochondria in single cells and determined whether nonirradiated mitochondria respond by undergoing $\Delta\Psi_m$ collapse. We used Rh123 for detection of $\Delta\Psi_m$ because this dye accumulates in mitochondria according to the Nernst equation and its emitted fluorescence (green; emission maximum at 529 nm) can be easily distinguished from that of CMXRos (red; emission maximum at 599 nm), thus allowing simultaneous imaging of both CMXRos and Rh123.

Human osteosarcoma cells loaded with Rh123 and CMXRos were subjected to continuous frame scanning (128 s), targeted by convention to the lower half of the cell. Such a high laser intensity typically resulted in photobleaching of both Rh123 and CMXRos in irradiated mitochondria (Fig. 1a, Fig. 1b). We interpret the loss of Rh123 fluorescence in both irradiated and nonirradiated mitochondria for the following reasons. Despite the possibility of photobleaching of Rh123 particularly in the irradiated zone, it was possible to detect early depolarization in such irradiated mitochondria, as evidenced by the faster decline in Rh123 when mitochondria were irradiated in the presence of CMXRos (Fig. 2a) than when CMXRos was absent (Fig. 2b). Specifically, quantification of the Rh123 intensity levels in the irradiated mitochondria at every 4 s during the irradiation revealed a decline in Rh123 intensity between 8 and 20 s since the start of irradiation, either with or without CMXRos (presumably due to photobleaching). However, in this brief time span, Rh123 intensity in mitochondria photoactivated with CMXRos was significantly lower than that of mitochondria in cells irradiated without CMXRos (Fig. 2b), reflecting the rapid depolarization in mitochondria due to CMXRos photosensitization. Prolonged laser scanning (after 32 s) resulted in substantial photobleaching of Rh123, as indicated by the reduction of Rh123 intensity in both cell groups irradiated with or without CMXRos, ultimately to similar levels (up to 128 s). The higher Rh123 intensity in mitochondria of cells irradiated with CMXRos after 36 s compared to their Rh123-loaded counterparts could be due to the CMXRos-induced release of the Rh123 from mitochondria into the cytosol, effectively unquenching some of the Rh123 trapped inside mitochondria and resulting in a higher fluorescence signal than cells not loaded with CMXRos. This increase in overall cellular Rh123 fluorescence following depolarization has also been observed in neurones treated with the protonophore uncoupler carbonyl cyanide m-chloro phenyl hydrazone (CCCP)[9].

Further evidence that the concomitant loss of Rh123 in nonirradiated mitochondria (Fig. 1a) was due to $\Delta\Psi_m$ collapse rather than photobleaching was obtained as follows. When cells loaded with Rh123 alone were partially irradiated (Fig. 1c), Rh123 was clearly retained in nonirradiated mitochondria. In these cells, diffusion of Rh123 from the unbleached nonirradiated zone to the bleached irradiated zone was not significant, as approximately 70% of the initial fluorescence was retained in the nonirradiated mitochondria (Fig. 2c inset). In contrast, partial irradiation in the presence of CMXRos resulted in a greatly reduced Rh123 intensity (Fig. 2c inset) to levels similar to those cells treated with CCCP to depolarize mitochondria.

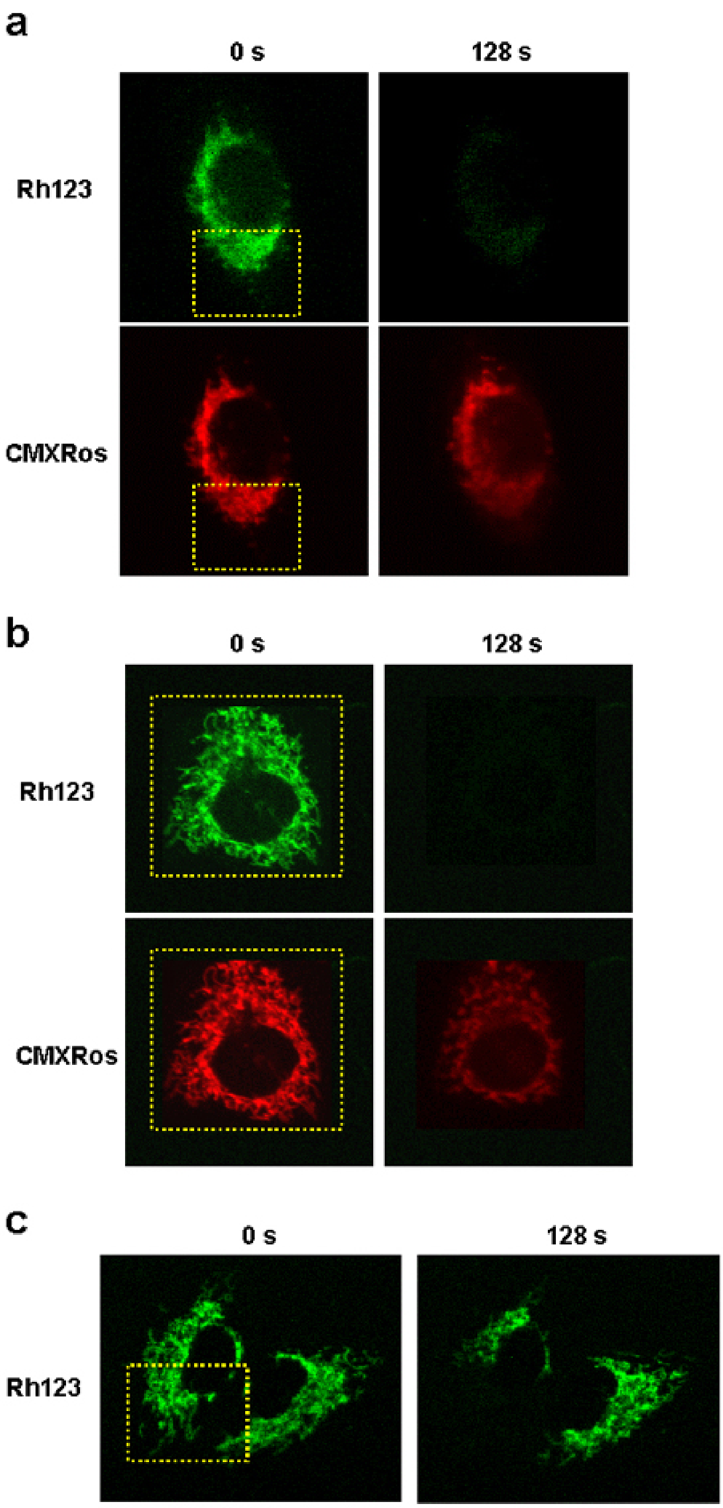

**FIGURE 1**. Rapid loss of $\Delta\Psi_m$ in cells subjected to partial or global irradiation using microscopic photosensitization. (a) Confocal imaging of human osteosarcoma 143B TK$^-$ cells before (0 s) and at end of partial irradiation (128 s) loaded with Rh123 (10 µM) and CMXRos (200 nM). Dashed box denotes the area of the cell subjected to confocal laser irradiation for 128 s. $\Delta\Psi_m$ response indicated by Rh123 is representative of 18 of 21 cells. (b) Imaging of cells subjected to global irradiation under same conditions as in (a). Loss of Rh123 signal in whole cell is representative of all 30 cells analyzed. (c) Control cells loaded with Rh123 were subjected to partial irradiation as in (a). Retention of Rh123 in nonirradiated mitochondria is representative of all 16 cells analyzed.

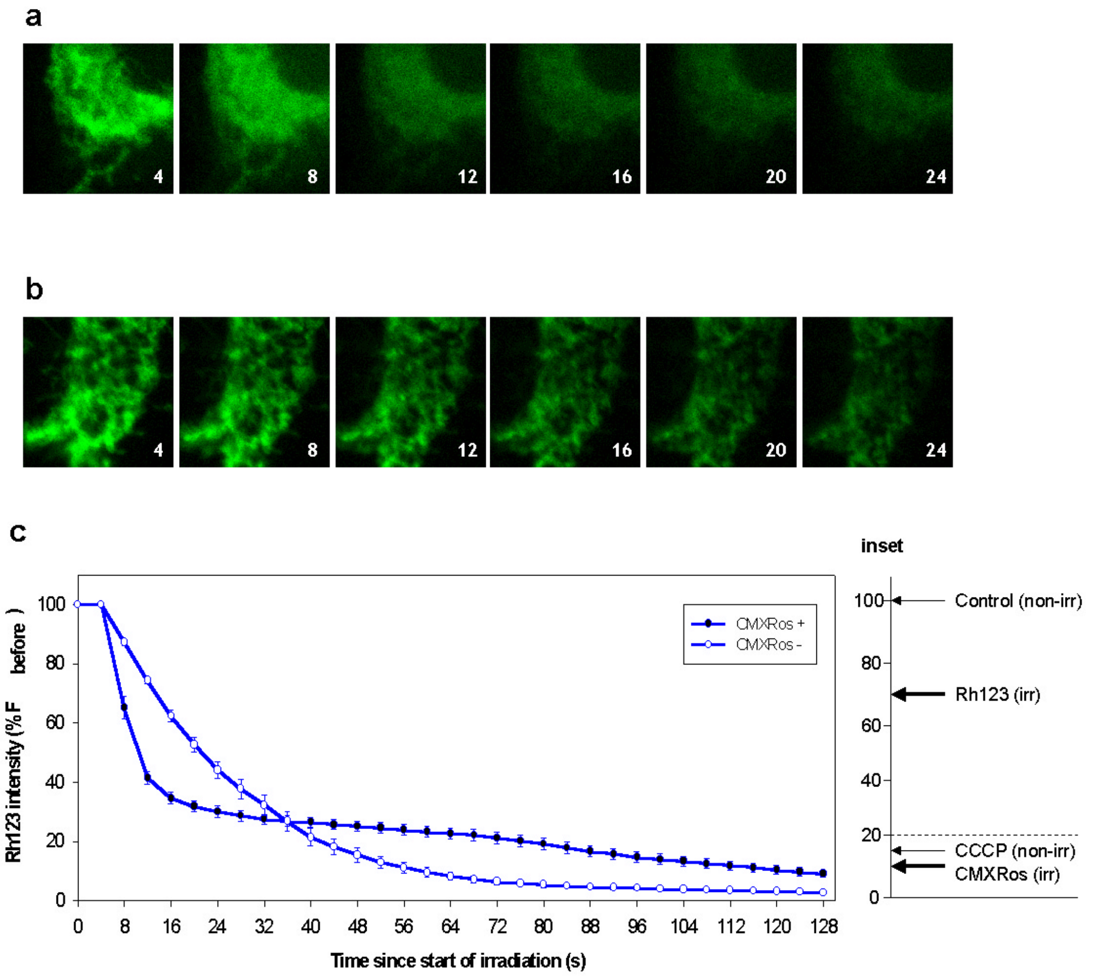

**FIGURE 2**. Changes in $\Delta\Psi_m$ during microscopic photosensitization under various conditions. Cells were imaged at different times during a 128 s series of intensive laser scans. (a) Confocal images showing loss of Rh123 fluorescence in irradiated mitochondria of a cell partially irradiated at the lower half. The cell was loaded with CMXRos (200 nM) and Rh123 (10 µM) prior to irradiation. (b) Rh123 fluorescence images of irradiated mitochondria in a cell loaded with only Rh123. (c) Quantification of the progressive loss of Rh123 fluorescence intensity in irradiated mitochondria of cells following laser irradiation. Control cells (to indicate loss of Rh123 due to photobleaching) were loaded with only Rh123 (n = 10). Other cells were loaded with Rh123 and CMXRos (n = 10). A region of interest in irradiated mitochondria from each cell was selected to determine the fluorescence intensity of Rh123 in pixel units. Rh123 intensity changes for each cell were obtained from stack images accumulated from the whole duration of irradiation, each image collected at every 4 s for a total of 128 s. The mean fluorescence intensity (pixel units ± S.E.M.) of Rh123 for each cell category at each time point was obtained by averaging the value obtained in 10 cells and expressed as a percentage of the initial fluorescence intensity ($F_{before}$). The inset (thick arrows) indicates Rh123 retention values in nonirradiated mitochondria of cells immediately after partial irradiation (irr) at 128 s or in all mitochondria of cells not irradiated (non-irr). Fluorescence values were 68% ± 5% (n = 13) for Rh123 alone or 11% ± 4% (n = 9) for Rh123 plus CMXRos. Control cells, loaded with Rh123 but not photoirradiated, were either further untreated or treated with CCCP (to indicate high or low $\Delta\Psi_m$, respectively). Fluorescence values were 100% ± 0% (n = 20) or 14% ± 2% (n = 15), respectively), thin arrows in inset. Dashed line represents the cutoff point for scoring cells either retaining (above line) or losing (below line) $\Delta\Psi_m$ in nonirradiated mitochondria after partial irradiation (as used in Fig. 3).

## Depolarization in all Nonirradiated Mitochondria is Contingent on a Critical Proportion of Mitochondria Photoirradiated

To understand further the nature of global $\Delta\Psi_m$ loss in nonirradiated mitochondria, it was important to determine what proportion of mitochondria needs to be photoirradiated in order to evoke global $\Delta\Psi_m$ loss. Partial irradiation on mitochondria at various proportions revealed an increasing tendency to lose $\Delta\Psi_m$ in nonirradiated mitochondria, up to the maximal extent when more than 45% of the mitochondria were irradiated (Fig. 3a; yellow bars). The data suggest that a certain degree of depolarization in irradiated mitochondria is required to initiate $\Delta\Psi_m$ collapse in nonirradiated mitochondria, as evidenced by the low percentage of cells showing $\Delta\Psi_m$ loss in nonirradiated mitochondria when <30% of mitochondria were irradiated (Fig. 3a). Moreover, the reduction in $\Delta\Psi_m$ response to partial irradiation at smaller proportions of mitochondria suggests that the laser irradiation effect was specific to the subset of mitochondria targeted.

In considering the loss of $\Delta\Psi_m$ in nonirradiated mitochondria, mitochondrial depolarization during cell death has been widely associated with the onset of MPT. This is characterized by an increase in the permeability of the inner mitochondrial membrane to solutes ($M_r < 1500$) due to the opening of a large conductance pore in the inner membrane. Whether MPT, as such, occurs in this case needs to be verified by further experimentation. If MPT is responsible for gross depolarization, one could envisage the recruitment of increasing fractions of mitochondria (close to the threshold potential for pore opening) to open their MPT pores as the intensity of the depolarization increases[10], which itself may induce full-blown MPT. In this model, a small or moderate depolarization following receipt by mitochondria of some signal (yet to be defined) is insufficient to result in MPT, whereas a more intense depolarization will lead to the opening of MPT pores in the mitochondrial population as a whole.

## $\Delta\Psi_m$ Loss in Nonirradiated Mitochondria Leads to an Apoptotic Outcome

We examined whether cells with $\Delta\Psi_m$ loss in nonirradiated mitochondria following partial irradiation undergo apoptosis. Cells loaded with CMXRos were irradiated to different extents in terms of the proportion of mitochondria exposed to laser scanning (as before), this time monitoring apoptotic outcomes (Fig. 3a). Cells subjected to either partial or global irradiation generally undergo apoptosis as demonstrated by the binding of annexin V to exposed phosphatidylserine (blue bars) on the outer leaflet of plasma membrane and cyt c release from mitochondria (black bars). Typical images of relevant cells are shown in Fig. 3b. No uptake of PI was observed (Fig. 3a; red bars) after partial irradiation, indicating that cells were undergoing early apoptosis. However, the progress toward cell death in partially irradiated cells was generally slower than that in globally irradiated cells. This was evidenced by the lack of cells showing PI uptake up to 2 h after partial irradiation, in contrast to a small percentage (close to 20%) of globally irradiated cells that lost plasma membrane integrity as indicated by PI uptake into the nucleus. Comparing the loss of $\Delta\Psi_m$ with apoptotic outcomes, irradiation of a smaller proportion of mitochondria (15–30%) minimized $\Delta\Psi_m$ loss in nonirradiated mitochondria but had less affect on apoptosis. In contrast, maximal $\Delta\Psi_m$ loss in nonirradiated mitochondria and apoptotic outcome was observed when partial irradiation was targeted to 45–65% of mitochondria. Taken together, these data demonstrate that damage to a subset of mitochondria is sufficient to induce a coordinated death response, but the role of nonirradiated mitochondria under conditions of restricted partial irradiation remains to be established.

An important finding is that partial irradiation (targeted to 46–55% of the mitochondria) initiated rapid cyt c release in a majority of the cells (more than 80%) following photoirradiation. In contrast, global irradiation resulted in an apparently lower percentage of cells showing cyt c release. This was detected in ~30% of cells after irradiation (Fig. 3a; black bar with asterisk). Some of the globally irradiated cells show apparent nonrelease of cyt c with weak punctate staining localized to mitochondria (data not shown). These anomalous observations of cyt c release following CMXRos photoirradiation may be due to degradation or conformational changes of cyt c that could avoid immunocytochemical detection subsequent to its initial

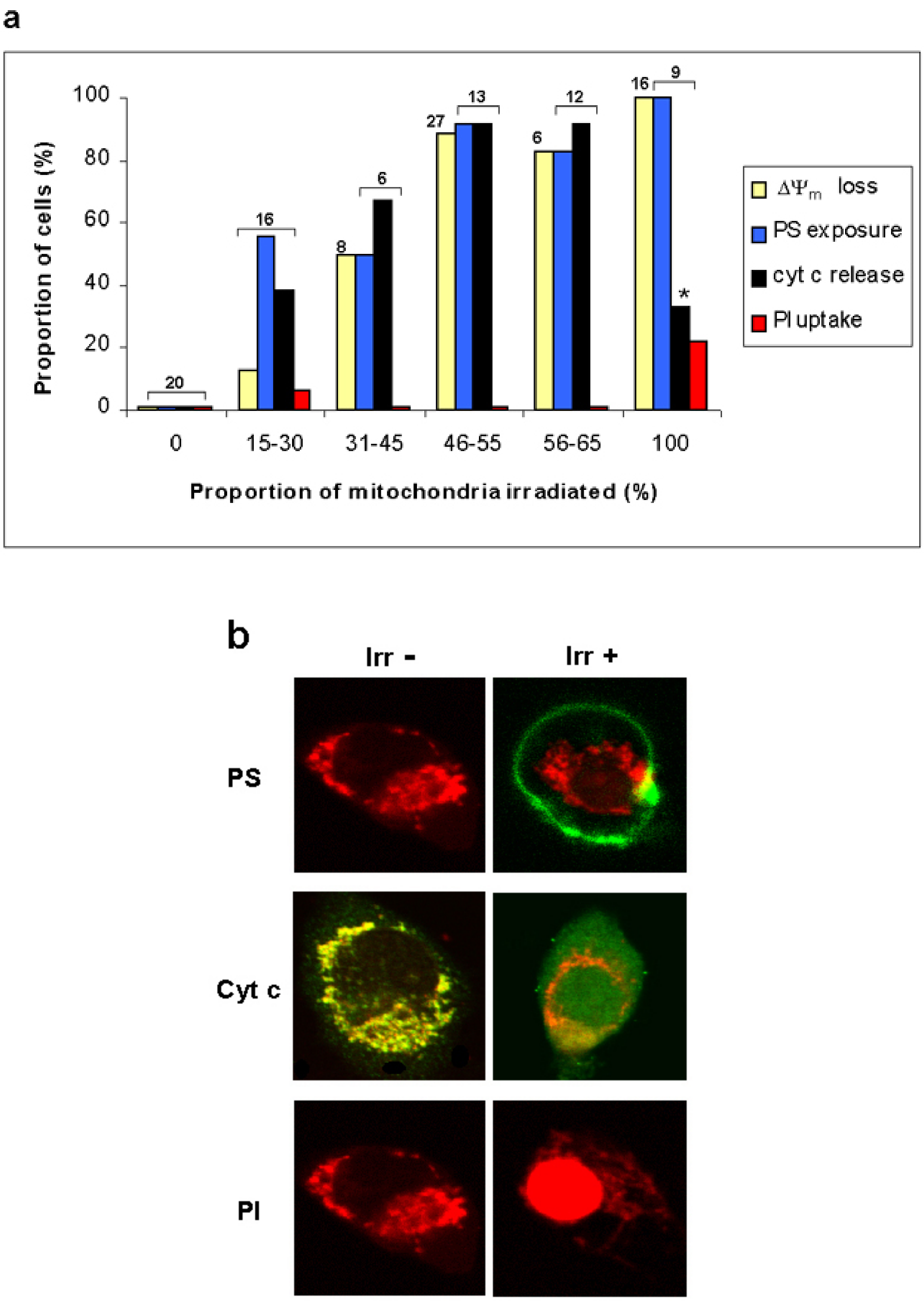

**FIGURE 3**. Mitochondrial dysfunction and death outcome following partial irradiation of various proportions of mitochondria irradiated in individual cells. (a) Proportion of cells showing immediate $\Delta\Psi_m$ loss in nonirradiated mitochondria of CMXRos-loaded cells following partial irradiation as detected by Rh123 (10 µM) and apoptotic outcomes as indicated by phosphatidylserine (PS) exposure, cyt c release, and lack of propidium iodide (PI) uptake. Quantification of the mitochondria irradiated was carried out as described in the Methods section. Loss of $\Delta\Psi_m$ was determined as in Fig. 2c. Other imaging aspects are detailed in the caption to Fig. 3b. Data for PS exposure, cyt c release, and PI uptake were collected at 45 min-2 h postirradiation. The number of cells analyzed in each category is indicated on top of the bars. An asterisk (*) reflects artifactual loss of cyt c immunostaining (see text). (b) Representative overlay confocal images showing control cells nonirradiated (Irr-; left column) and partially irradiated cells (Irr+; right column) with a death outcome. Cells loaded with CMXRos (red) were stained with either annexin V-Alexa Fluor 488 (green; top row) to detect PS exposure, anti-cyt c antibody coupled to Alexa Fluor 488 (green; middle row) to detect cyt c release or PI (red; bottom row) for determining plasma membrane integrity. Colocalization of red with green appears yellow. PS exposure detected by Annexin V binding to plasma membrane is depicted by a green ring (top right image). Cyt c release is characterized by diffuse cyt c fluorescence (green) in the whole cell (middle right image). Irradiated cells with permeabilized plasma membrane show red nuclear staining of PI (bottom right), indicating necrosis; the majority of irradiated cells are apoptotic and appear as in the bottom left panel.

release[11]. The residual weak staining in mitochondria could reflect the remaining proportion of unreleased cyt c bound to the mitochondria that was not removed following permeabilization of the cellular membranes with Triton-X-100 during work-up of the fixed cells prior to immunocytochemical staining. In our case, such loss of immunochemical responsiveness of cyt c could arise from (a) direct photodamage on mitochondria or (b) a burst of reactive oxygen species consequent on photodynamic action of CMXRos. In any case, this finding emphasizes the practical advantages of irradiation under controlled conditions (partial, rather than global) as shown under the partial irradiation conditions used here.

## Microscopic Photosensitization as a New Tool in Studying Mitochondria and Cell Death

The technique of microscopic photosensitization has been set up optimally to induce apoptosis in a subpopulation of mitochondria in single cells. This cannot be achieved by use of drugs, genomic mutations, or overexpressed gene products that obligatorily affect all mitochondria of any given cell. The partial-irradiation approach enables direct investigation of intermitochondrial signaling. The details of this process can now be analyzed at several integrated levels, achieved by using various fluorescent indicators (exogenous labeling with fluorescent dyes or intrinsic labeling of proteins tagged to green fluorescent protein) specific for monitoring subcellular events in nonirradiated mitochondria. We postulate that a signal emanates from irradiated to nonirradiated mitochondria by loss of $\Delta\Psi_{m.}$ Investigations are required to determine the basis for $\Delta\Psi_m$ dissipation in nonirradiated mitochondria. Under our partial-irradiation conditions, $Ca^{2+}$ seems to be an attractive candidate for consideration in this respect, as $Ca^{2+}$ overload in the mitochondria can readily induce mitochondrial depolarization, usually via an MPT mechanism[3]. $Ca^{2+}$ has also been recognized as the means whereby waves of mitochondrial depolarization travel along the relatively large myotubes[6].

Moreover, it remains to be resolved whether during the execution of apoptosis irradiated mitochondria also activate the nonirradiated mitochondria to release cyt c. This will shed light on whether cyt c is employed as a signal amplifier or trigger in the execution of apoptosis and whether localized release of apoptogenic proteins in a small region of the cell is enough to engage the cell into the apoptotic pathway. Released cyt c from mitochondria photodamaged by other mitochondria-selective photosensitizers has been shown to directly initiate apoptosis via caspase-3 activation[12] or indirectly by another pathway involving caspase-8-mediated feedback amplification of cyt c release in certain cells[13].

## ACKNOWLEDGMENTS

We thank the TheScientificWorld editor responsible for handling the manuscript.

## BIOSKETCHES

**May Ghee Lum** is a Graduate student (Ph.D), Department of Biochemistry and Molecular Biology, Monash University. She holds a BSc in Biotechnology (Hons). Her honours work involved the construction of chimeric proteins that are potentially novel microbial cellulases with application in large-scale fermentation. Currently, her doctoral research is on mitochondrial regulation in the context of cell death in mammalian systems. A key finding from this work is the demonstration of signaling between mitochondria as a subcellular response to induction of apoptosis. This was investigated by using microscopic photosensitization, a novel technique she developed in the laboratory to induce damage on a subset of mitochondria in any given cell. Signaling between mitochondria was indicated by a response in the non-irradiated mitochondria. Ms. Lum received the Wood-Whelan Research Fellowship (The International Union of Biochemistry and Molecular Biology) in 2001 to visit the laboratory of Dr. Douglas Green at the La Jolla Institute for Allergy and Immunology in San Diego, and a Monash University postgraduate travel grant to attend the 2001 Miami Nature Biotechnology Winter Symposium.

**Tetsuhiro Minamikawa** is an Application Specialist, Scientific Equipment Division, Olympus Australia Pty. Ltd. He holds M.D. and Ph.D. degrees and is a Registered Pathologist (Japanese Pathological Society). With the background of cardiology and pathology, Dr. Minamikawa's research interests initially included cellular and molecular mechanisms of myocardial infarction and cardiomyopathy. His research team was also one of the first in the world to develop biological confocal microscopy and UV confocal system in late 1980s to early 1990s. His research interests have shifted toward the advanced live-cell imaging techniques and, as such, he currently is involved in a consultant position for imaging applications. He received the University Award for outstanding publications (Kyoto Prefectural University of Medicine) in 1993 for a paper titled, "Confocal Calcium Imaging with Ultraviolet Laser-Scanning Microscopy and Indo-1".

**Phillip Nagley** is Professor of Biochemistry (Personal Chair), Department of Biochemistry and Molecular Biology, Monash University. He holds B.Sc. (Hons), M.Sc., Ph.D., and D.Sc. degrees. Dr. Nagley's research interests encompass the molecular and cellular biology of mitochondria in a range of contexts. Current work involves utilisation of the fluorescence properties of proteins tagged with GFP and its variants, to study in novel ways the association of protein subunits within ATP synthase. His interest in molecular biology of ageing involves characterisation and quantification of age-associated mtDNA mutations in mammalian tissues, drawing links between accumulation of mtDNA mutations and the age-associated decline in physiological function. His studies on mitochondrial regulation in relation to cell death are applied to cultured cells including neurons. Current research investigates release of cytochrome *c* from mitochondria and communication between different mitochondria of the same cell during apoptotic signaling. Dr. Nagley was awarded the University Medal in Biochemistry (University of Sydney) in 1968, the Boehringer-Mannheim Medal (Australian Biochemical Society) in 1978, and the Lemberg Medal (Australian Society for Biochemistry and Molecular Biology) in 2001.
